# Increased Frequency of the Non-Dipper Blood Pressure Pattern in Patients with Systemic Sclerosis: Insights from 24-Hour Ambulatory Monitoring

**DOI:** 10.3390/jpm15060253

**Published:** 2025-06-15

**Authors:** Oğuzhan Zengin, Gülşah Soytürk, Burak Göre, Mustafa Yürümez, Ali Can Kurtipek, Emra Asfuroğlu Kalkan, Hatice Ecem Konak, Şükran Erten, Ihsan Ateş

**Affiliations:** 1 Department of Internal Medicine, University of Health Sciences, Ankara Bilkent City Hospital, 06800 Ankara, Turkey; ihsan.ates@sbu.edu.tr; 2 Department of Rheumatology, Ankara Bilkent City Hospital, 06800 Ankara, Turkey; gulsah.soyturk@saglik.gov.tr (G.S.); haticeecemkonak@gmail.com (H.E.K.); sukranerten@aybu.edu.tr (Ş.E.); 3 Department of Internal Medicine, Çerkeş State Hospital, 16800 Çankırı, Turkey; burak.gore@sbu.edu.tr; 4 Department of Internal Medicine, Ankara Bilkent City Hospital, 06800 Ankara, Turkey; mustafayurumez@gmail.com (M.Y.); emra.asfuroglukalkan@sbu.edu.tr (E.A.K.); 5 Department of Internal Medicine, Faculty of Medicine, Ankara University, 06230 Ankara, Turkey; ackurtipek@ankara.edu.tr

**Keywords:** blood pressure monitoring, ambulatory, blood pressure, circadian rhythm, scleroderma, non-dipper pattern

## Abstract

**Background:** In systemic sclerosis (SSc), endothelial dysfunction, inflammation, and reduced nitric oxide levels may disrupt circadian blood pressure (BP) regulation. There are studies showing that inflammatory and certain other cells in diseases like SSc exhibit diurnal rhythms. In our study, we examined the effect of SSc on BP. In particular, the frequency of the non-dipper pattern (lack of nighttime BP reduction) in SSc patients has not been adequately investigated. The aim of this study was to evaluate the 24 h BP profile in SSc patients and to compare the frequency of the non-dipper pattern with that of the non-scleroderma group. Additionally, the identification of disrupted circadian BP patterns in SSc patients aims to contribute to the development of personalized, time-sensitive BP monitoring strategies in the future and to support the applicability of personalized medicine in this context. **Methods:** A total of 31 SSc patients diagnosed according to the 2013 ACR/EULAR classification criteria and 30 age- and sex-matched individuals without SSc were included in this prospective study. BP changes between day and night were evaluated by measuring BP every 30 min with a 24 h ambulatory blood pressure monitoring (ABPM) device. The non-dipper pattern was defined as a decrease in BP of less than 10% during the night compared to the day. To better assess BP fluctuations during the night, nighttime measurements were divided into two time periods: first, 24:00–04:00, and then 04:00–08:00. Additionally, laboratory and clinical parameters and SSc subtypes were compared between the groups. **Results:** The ABPM findings were compared between the groups with and without SSc. The non-dipper pattern was significantly more common in the SSc group at all time intervals. The non-dipper pattern was observed in 25.8% of the non-SSc group and 83.9% of SSc patients (*p* < 0.001). In the period between 24:00 and 04:00, the prevalence was 25.8% in the control group and 71.0% in SSc patients (*p* < 0.001), and between 04:00 and 08:00, it was 35.5% in the control group and 80.6% in SSc patients (*p* < 0.001). No significant difference was found in non-dipper patterns between individuals with diffuse and limited cutaneous forms of systemic sclerosis. **Conclusions:** The non-dipper BP pattern is significantly more common in patients with SSc, indicating the disruption of the circadian rhythm affecting BP. Analysis performed by dividing the night into specific time periods revealed that this deterioration continued throughout the night. The findings highlight the importance of circadian BP monitoring in SSc patients and may contribute to future risk stratification and treatment strategies. Circadian BP analysis in SSc may help to develop strategies that are personalized for these patients and tailored to their physiological rhythm.

## 1. Introduction

Persistent inflammation adversely affects vascular endothelial integrity, fostering the development of atherosclerotic plaque and contributing to increased arterial rigidity. This disturbance hampers normal hemodynamics and leads to elevated blood pressure [1]. Plaque buildup within the vascular walls results in multiple changes to the endothelial performance. Inflammatory pathways further exacerbate vascular injury by promoting the synthesis of cytokines, signaling agents, and reactive oxygen species [2]. Such inflammatory activity elevates the likelihood of hypertension (HTN), disrupting cardiovascular homeostasis and potentially triggering significant health complications [3]. Autoimmune diseases, such as systemic sclerosis (SSc), play a significant role in the development of HTN. Conditions like SSc are associated with vascular damage, endothelial dysfunction, and extensive tissue fibrosis. Damage to the endothelial layer reduces the ability of blood vessels to dilate and contract properly. Similarly, vascular inflammation and fibrotic changes compromise the structural integrity of the vessels. These pathologic mechanisms in the vascular system contribute to increased BP and are key factors in the onset of HTN [4,5].

Various parameters of the immune system vary depending on the circadian rhythm. This circadian rhythm affects many characteristics of immune system cells, from their activity to their functions, leading to temporal changes in their responses [6]. Low-grade chronic inflammation that emerges with aging leads to disruptions in the immune functions regulated by the biological clock [7]. This process may play a role in the mechanisms of inflammatory diseases such as SSc. The biological clock plays a crucial role in immune system function by regulating the circadian rhythm of immune cell trafficking and inflammatory responses. In particular, changes in the levels of neuroendocrine mediators such as epinephrine and cortisol during the sleep–wake cycle control the circulation of immune cells and their homing to lymph nodes, thereby optimizing the timing of adaptive immune responses [8].

Although previous studies have suggested that circadian variations in immune regulators may influence vascular tone, our study did not directly assess immune parameters. Recent studies have shown that the immune system plays a role in the pathogenesis of hypertension (HTN), particularly through the Th17 cell pathway. Th17 cells are critical for the production of inflammatory cytokines, especially IL-17, and these cytokines may contribute to the development of HTN by promoting inflammation in the vascular walls. Animal studies have demonstrated that high salt levels activate the immune system by stimulating Th17 cells and increasing the production of IL-17 and other pro-inflammatory cytokines [9].

Prolonged inflammation leads to the overproduction of reactive oxygen products and creates oxidative stress. This biological response causes the development of endothelial dysfunction. Endothelial dysfunction and inflammation cause a decrease in the effect of nitric oxide, resulting in a decrease in the vasodilator role of this molecule and its ability to relax blood vessels. Additionally, inflammation stimulates T cells through the adaptive immune system, leading to the release of cytokines that promote vasoconstriction. As a result, long-term inflammation and the resulting oxidative stress, activation of the immune system, and damage to the vascular walls accelerate the development of cardiovascular diseases such as HTN [10]. The combination of these mechanisms suggests that SSc disease constitutes an important risk factor for HTN and circadian rhythm disruption. Systemic sclerosis may contribute to cardiovascular disease development by causing long-term alterations in immune system activity and vascular function.

The correlation between circadian blood pressure (BP) patterns and cardiovascular diseases is already known. Notably, the non-dipper pattern is associated with a higher risk of HTN [11]. Scleroderma, also known as systemic sclerosis, is a connective tissue disorder that predominantly affects women of a reproductive age and is marked by fibrosis and multi-organ involvement. Clinically, scleroderma is classified into three primary phenotypes: limited cutaneous systemic sclerosis (lcSSc), systemic disease, and overlap syndrome [12]. Previously referred to as CREST syndrome, lcSSc is characterized by localized skin thickening, primarily affecting distal regions such as the elbows and knees. While organ involvement is observed less frequently in these patients, the incidence of pulmonary hypertension is higher [13]. Diffuse scleroderma presents with Raynaud’s phenomenon, swollen hands, fatigue, and joint pain. In this form, skin thickening can extend beyond distal areas such as the elbows and knees, affecting the proximal extremities. Fibrosis may develop in organs, and this can seriously impair patients’ organ functions. Diffuse scleroderma has a significant impact on cardiovascular and pulmonary functions and is generally associated with worse prognosis [14].

Systemic sclerosis is a disease characterized by multi-organ involvement and serious vascular complications. The use of corticosteroids, especially at high doses, can increase the risk of scleroderma renal crisis; therefore, caution is advised in patients with early diffuse forms. The 2024 British Society for Rheumatology guideline emphasizes the comprehensive and multidisciplinary management of organ damage and complications in systemic sclerosis. In this context, personalized and multifaceted treatment approaches are essential for patients [15,16].

HTN is the most common preventable risk factor of all-cause mortality and affects approximately 1.4 billion adults worldwide [17]. Normally, BP exhibits a certain rhythm throughout the day; it increases in the morning hours, stabilizes at some level throughout the day, and at night, a decrease of 10–20% from the average daytime BP is observed. Normally, BP drops at night, called the “dipper pattern”. However, in some patients, this decrease is not observed during the night hours; this is called the “non-dipper pattern” and is associated with a more severe form of HTN and cardiovascular complications. Nocturnal SBP ≥ 120 mmHg and DBP ≥ 70 mmHg, measured using a 24 h ambulatory blood pressure monitoring (ABPM) device, and daytime BP < 135/85 mmHg, defining isolated nocturnal HTN. Nocturnal HTN and the non-dipper rhythm increase the risk of developing HTN-induced organ damage. Circadian rhythms are regulated through the central clock governed by the suprachiasmatic nucleus of the hypothalamus and peripheral clocks located throughout the body [18,19]. However, the physiological mechanisms underlying non-falling and extremely high nocturnal BP are still not fully understood. The diffuse and limited cutaneous subtypes of systemic sclerosis exhibit distinct clinical courses, patterns of organ involvement, and serological features. As a result, vascular abnormalities, endothelial dysfunction, inflammation, and decreased nitric oxide levels in systemic sclerosis (SSc) patients can disrupt circadian BP regulation. It is well established that inflammatory cells exhibit diurnal rhythms in inflammatory diseases. In this study, the effects of SSc, as an inflammatory disease, on circadian BP were investigated. However, the relationship between SSc and circadian BP variations, particularly the frequency of the non-dipper pattern, has been studied in limited depth. The aim of this study was to examine the 24 h BP profile in SSc patients and compare the frequency of the non-dipper pattern with that of a non-scleroderma patient group. Furthermore, this study aimed to reveal circadian blood pressure abnormalities in SSc patients and contribute to the development of more precise approaches that will shape personalized diagnostic and treatment decisions in the future, thereby supporting the implementation of personalized medicine strategies.

## 2. Materials and Methods

This study was conducted prospectively at Ankara Bilkent City Hospital and included 31 SSc patients and 30 individuals without SSc. Circadian blood pressure measurements of the participants were performed using a 24 h ABPM device. The ABPM device measured the participants’ BP every 30 min, providing a total of 24 h of monitoring and allowing for the evaluation of BP changes between day and night. The non-dipper pattern was defined as a decrease in nighttime BP of less than 10% compared to the daytime. The study was approved by the Ankara Bilkent City Hospital Ethics Committee, and all procedures adhered to the ethical guidelines of the Declaration of Helsinki and its later revisions (Date: 18 September 2024, Decision No: TABED 2-24-409).

Systemic sclerosis diagnosis was made according to the classification criteria determined by the American College of Rheumatology (ACR) and the European League Against Rheumatism (EULAR) in 2013. In this study, pulmonary hypertension (PHT) assessment was performed based on the 2022 Pulmonary Hypertension Guidelines published by the European Society of Cardiology (ESC) and the European Respiratory Society (ERS). According to the ESC/ERS 2022 guideline, PHT can only be diagnosed via right heart catheterization. The diagnostic criteria include the mean pulmonary artery pressure (mPAP) ≥ 20 mmHg, pulmonary vascular resistance (PVR) ≥ 2 wood units, and pulmonary capillary wedge pressure (PAWP) ≤ 15 mmHg. Since right heart catheterization was not performed in our study, PHT suspicion was evaluated based only on echocardiography findings.

The transthoracic echocardiography (TTE) findings were analyzed based on the records obtained in the 12 months before the study inclusion date. In the evaluation of PHT suspicion, the tricuspid regurgitation flow velocity (TR Vmax) and accompanying structural/morphological findings were taken into consideration. In accordance with the ESC/ERS 2022 guideline, it was considered that the probability of PHT should be assessed based not only on the TR Vmax, but also on the presence of at least two supporting echocardiographic findings (e.g., right ventricular dilatation, interventricular septum flattening, right atrial enlargement, pulmonary artery dilatation). Accordingly, patients with a TR Vmax > 2.8 m/s and at least two concurrent supportive findings were classified as “PHT present”; patients who did not meet these criteria were evaluated as “PHT not present”. This classification is based solely on echocardiographic data, and the study does not include invasive hemodynamic measurements such as mPAP, PVR, and PAWP. Therefore, the results were interpreted based on suspicion of PHT rather than diagnosis, in line with the echocardiographic risk assessment principles of the ESC/ERS 2022 guideline.

Although capillaroscopy is an important method for demonstrating microvascular changes in SSc, it could not be performed in this study due to technical and logistical reasons. The presence of Reynaud’s phenomenon, digital ulcers, and pitting scars was assessed through physical examination and patient files. While Reynaud’s phenomenon was present in some patients, digital ulcers and pitted scars were not detected.

Renal involvement was evaluated according to clinical and laboratory criteria. No clinical signs or laboratory abnormalities suggestive of scleroderma renal crisis were observed in any patient during the study period. The serum creatinine levels of all patients were within the normal limits; no newly diagnosed hypertension, proteinuria, or hematuria was encountered. Therefore, no renal system involvement was present in any patient included in the study.

Pulmonary involvement, especially interstitial lung disease (ILD), was evaluated via high-resolution thoracic computed tomography (HRCT). The treatment information of systemic sclerosis patients was obtained from patient files. The use of mycophenolate mofetil (MMF), azathioprine, corticosteroids, hydroxychloroquine, phosphodiesterase-5 inhibitors (PDE5is), endothelin receptor antagonists (ERAs), and angiotensin receptor blockers (ARBs) has been noted. These drugs were planned individually by rheumatologists before the study, based on the patients’ immunological profile, organ involvement, and clinical condition. None of the patients in our study were receiving more than 1 mg/kg of steroids.

The severity of skin involvement was assessed using the Modified Rodnan Skin Score (mRSS). This method involves evaluating the skin thickness through palpation in 17 anatomical areas of the patient: face, chest, abdomen, upper arms, forearms, hands, thighs, legs, and feet. Each region was scored from 0 (normal) to 3 (severe thickness), and the total score was calculated to a maximum of 51. This semiquantitative assessment is widely used in clinical research to determine the severity of skin fibrosis in SSc.

A power analysis was performed using G*Power software for sample size calculation. In this analysis, it was evaluated whether the sample size was sufficient to detect the primary effects of the study. The analysis was performed considering a 95% confidence interval and 80% test power. The estimated difference (effect size) was taken as 0.5, which was accepted as representing a medium-sized effect. According to the results of the power analysis, a minimum of 28 participants were determined for each group. This sample size ensures that the statistical power is sufficient for detecting significant differences.

Individuals in the non-SSc group consisted of individuals in the same age range and with similar gender distribution. Some exclusion criteria were determined in our study: individuals under 18 years of age, those diagnosed with malignancy, individuals with incomplete study data, those with a history of renal artery stenosis or renal vein thrombosis, those diagnosed with chronic kidney disease, and patients diagnosed with resistant HTN despite treatment. These exclusion criteria were determined to ensure the homogeneity of the study and to increase the reliability of the results.

### 2.1. Blood Pressure Measurement

For this evaluation, ABPM was carried out using Suntech Bravo 222B devices, SunTech Medical Inc., Morrisville, NC, USA. Blood pressure measurements were taken every 30 min throughout a full 24 h cycle, ensuring at least 16 readings during daytime hours and no fewer than 6 during the night. The cuff was placed on the participant’s non-dominant arm. The collected data were examined using specialized software for this analysis. The mean BP values over a 24 h period were determined by averaging the systolic (SBP) and diastolic (DBP) readings from hourly measurements taken throughout both the day and night. A “dipper pattern” was identified if there was a decrease in nighttime BP greater than 10%, while a “non-dipper pattern” was observed if the decrease was less than 10%.

To assess the changes in nighttime BP, the measurements were divided into two separate 4 h intervals (00:00–04:00 and 04:00–08:00), aimed at evaluating the impact of elevated stress hormones and circadian rhythm. Each set of nighttime measurements was compared with the daytime data to determine if participants followed a dipper or non-dipper pattern. The Suntech Bravo 222B device used in this study was clinically tested and approved by the manufacturer, ensuring its accuracy and reliability for ambulatory blood pressure monitoring in different patient groups. Additionally, the device was calibrated before data collection began.

### 2.2. Statistical Analysis

In this study, the demographic characteristics and comorbidities between the control and patient groups were compared using the Chi-squared test. Age differences between the groups were analyzed using Student’s *t*-test, as the data followed a normal distribution. For other continuous variables (such as hemoglobin, creatinine, potassium, etc.), the Mann–Whitney U test was applied due to the non-normal distribution of the data. In the comparison of laboratory parameters, Student’s *t*-test was used for parametric data, and the Mann–Whitney U test was applied for non-parametric data. A *p*-value of <0.05 was considered statistically significant for all comparisons. Ambulatory blood pressure measurements were compared using both Student’s *t*-test and the Mann–Whitney U test. Furthermore, the Mann–Whitney U test was used to examine blood pressure changes over time periods. Finally, the comparison of dipping patterns (nighttime–daytime blood pressure reduction) and serological parameters was performed using the Chi-squared test. All analyses were conducted using SPSS (SPSS version 26.0 IBM Corp., Armonk, NY, USA).

## 3. Results

Table 1 presents the demographic and clinical characteristics of the SSc and non-SSc groups, including age, gender distribution, and presence of hypertension. There were no statistically significant differences between the groups in any of these variables. The mean age was 52.2 ± 11.1 years in the non-SSc group and 55.4 ± 10 years in the SSc group (*p* = 0.234). The proportion of female participants was 87.1% in the non-SSc group and 93.5% in the SSc group (*p* = 0.688). The prevalence of hypertension was 32.3% in the non-SSc group and 35.5% in the SSc group, with no significant difference observed (*p* = 0.788). Additionally, the BMI values were found to be similar between the groups, with a mean BMI of 25.8 ± 3.9 kg/m^2^ in the non-SSc group and 26.1 ± 4.2 kg/m^2^ in the SSc group (*p* = 0.762). These findings indicate that the two groups were demographically and clinically comparable, minimizing the potential influence of these variables on the study outcomes.

Table 2 presents the comparison of laboratory parameters between the SSc and non-SSc groups. Statistically significant differences were observed in hemoglobin levels, leukocyte counts, and potassium levels. The mean hemoglobin level was significantly lower in the SSc group (12.0 g/dL) compared to the non-SSc group (14.0 g/dL) (*p* < 0.001). Leukocyte counts were also significantly lower in the SSc group (5.74 × 10^9^/L) compared to the non-SSc group (7.3 × 10^9^/L) (*p* = 0.002). Potassium levels were significantly lower in the SSc group (4.3 mEq/L) compared to the non-SSc group (4.5 mEq/L) (*p* = 0.013).

Other laboratory parameters, such as creatinine, sodium, liver enzymes, and lipid profiles, showed no significant differences between the two groups (*p* > 0.05). These findings indicate that while some laboratory parameters differ significantly between the groups, many others remain similar, suggesting a relatively comparable laboratory profile between the SSc and non-SSc groups.

Table 3 presents the comparison of ABPM data between the systemic sclerosis (SSc) and non-SSc groups. Statistically significant differences were found in daytime diastolic and 24 h diastolic BP measurements. The daytime DBP was significantly lower in the SSc group (68 mmHg) compared to the non-SSc group (76 mmHg) (*p* = 0.014). Similarly, the 24 h DBP was also significantly lower in the SSc group (67 mmHg) compared to the non-SSc group (71 mmHg) (*p* = 0.031). Those written in bold are statistically significant.

No significant differences were observed in SBP measurements, whether during the daytime, nighttime, or over the 24 h period. The nighttime diastolic pressure (*p* = 0.086) and blood pressure measurements during the 24:00–04:00 and 04:00–08:00 periods did not show significant differences between the two groups (*p* > 0.05). Those written in bold are statistically significant.

These findings suggest that while there are notable differences in diastolic blood pressure values between the SSc and non-SSc groups, the systolic blood pressure measurements do not differ significantly, indicating a relatively comparable blood pressure profile between the two groups across various time periods.

**Figure 1:**
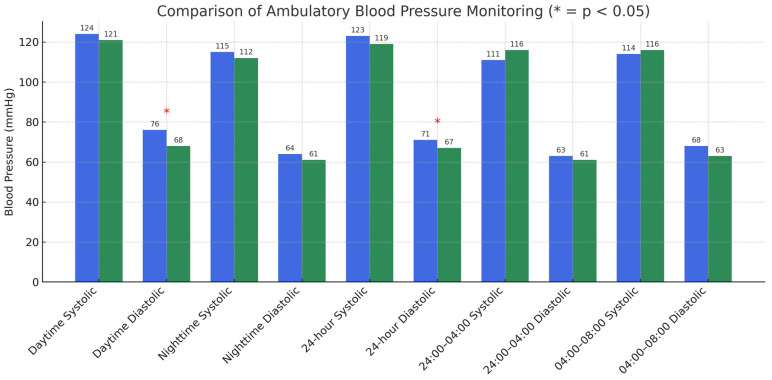
Ambulatory blood pressure measurements (median) of systemic sclerosis (SSc) and non-SSc groups. The blue bars represent the non-scleroderma patient group, whereas the green bars represent the systemic sclerosis (SSc) patient group.

Figure 1 illustrates the ambulatory blood pressure measurements (median values) for both the SSc and non-SSc groups.

Table 4 presents a comparison of the non-dipping patterns between the SSc and non-SSc groups across different time intervals. The frequency of non-dipping was significantly higher in the SSc group across all time periods.

During the day–night interval, 83.9% of SSc patients exhibited a non-dipper pattern compared to 25.8% in the non-SSc group (*p* < 0.001). Similarly, in the 24:00–04:00 interval, non-dipping was observed in 71.0% of the SSc group versus 25.8% of the non-SSc group (*p* < 0.001). In the 04:00–08:00 interval, the non-dipper frequency was 80.6% in the SSc group and 35.5% in the non-SSc group (*p* < 0.001).

When the time intervals were compared within each group, no statistically significant differences were observed in the non-dipping frequency (non-SSc group: *p* = 0.778; SSc group: *p* = 0.437), indicating that the distribution of non-dipper patterns remained consistent across the evaluated time periods within both groups.

These findings highlight a significantly higher prevalence of non-dipping BP patterns in patients with systemic sclerosis, particularly during the nighttime periods.

**Figure 2:**
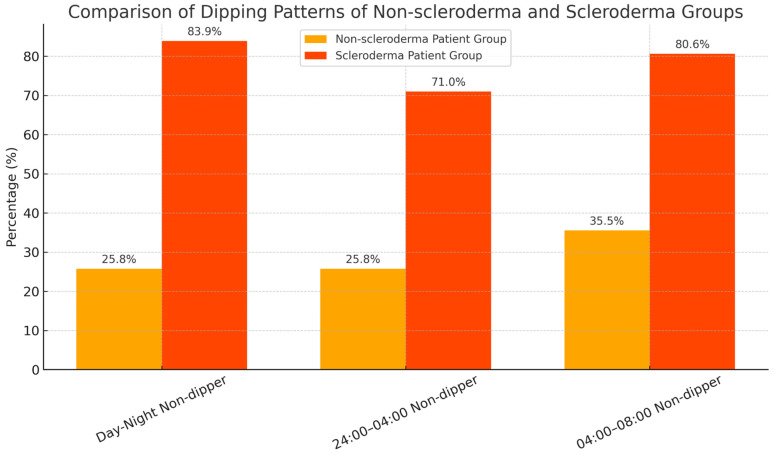
Comparison of the frequency of the non-dipper patterns between systemic sclerosis and non-systemic sclerosis patient groups.

Figure 2 shows the comparison of the frequency of the non-dipper patterns between systemic sclerosis and non-systemic sclerosis patient groups.

Table 5 summarizes the comparative clinical, serological, and treatment-related characteristics of patients with diffuse cutaneous systemic sclerosis and limited cutaneous systemic sclerosis. In terms of circadian blood pressure regulation, no statistically significant differences were observed between the two groups. During the day–night interval, 88.2% of patients with diffuse cutaneous systemic sclerosis and 78.6% of those with limited cutaneous systemic sclerosis exhibited a non-dipper blood pressure pattern (*p* = 0.467). Similar non-significant trends were found in the 24:00–04:00 and 04:00–08:00 intervals (*p* = 0.457 and *p* = 0.239, respectively). However, there were notable differences in the autoantibody profiles. Anti-SCL-70 (topoisomerase I) positivity was significantly higher in patients with diffuse cutaneous involvement (76.5%) compared to those with limited cutaneous involvement (7.1%) (*p* < 0.001). Conversely, Anti-centromere B antibody was detected exclusively in the limited group (78.6%) and completely absent in the diffuse group (*p* < 0.001). Although not statistically significant, Anti-RNP/Sm and Anti-SS-A antibodies were observed more frequently in the diffuse group (*p* = 0.052 and *p* = 0.098, respectively). Interstitial lung disease was significantly more common in the diffuse cutaneous group, affecting 70.6% of patients versus 21.4% in the limited group (*p* = 0.006). No significant differences were observed in the modified Rodnan skin score or pulmonary hypertension prevalence between the two subtypes. When treatment profiles were compared, the use of angiotensin receptor blockers and mycophenolate mofetil was significantly higher in the diffuse group (*p* = 0.015 and *p* = 0.025, respectively), likely reflecting greater disease severity and systemic involvement. Although not statistically significant, phosphodiesterase-5 inhibitors were also used more frequently in the diffuse group (35.3% vs. 7.1%, *p* = 0.062), which may relate to their pulmonary vasodilatory effects in patients with more extensive vasculopathy. The use of calcium channel blockers and hydroxychloroquine was common in both groups, without significant differences.

Table 6 illustrates the comparative distribution of serological markers, clinical manifestations, and therapeutic regimens between systemic sclerosis patients categorized by their 24 h ambulatory blood pressure profiles as either day–night dippers (n = 5) or non-dippers (n = 26). The analysis revealed no statistically significant differences in the distribution of key autoantibodies—including anti-topoisomerase I, anti-centromeric proteins, anti-RNA polymerase, and anti-Ro-52—between the two groups, suggesting that the serological profiles were largely comparable across dipping status.

Interstitial lung disease and pulmonary hypertension, two major organ involvements in systemic sclerosis, were also similarly distributed between dippers and non-dippers (interstitial lung disease: 40% vs. 50%, *p* = 0.682; pulmonary hypertension: 60% vs. 53.8%, *p* = 0.800). These findings indicate that circadian blood pressure variation was not significantly associated with the extent of pulmonary or vascular involvement.

In terms of immunosuppressive therapy, although not statistically significant, mycophenolate mofetil use was more frequent in the non-dipper group (42.3%), possibly reflecting a higher rate of active or progressive disease requiring intensive management. Conversely, azathioprine use was more commonly observed in the dipper group (60% vs. 19.2%, *p* = 0.056), suggesting a potential treatment trend difference. Hydroxychloroquine, corticosteroids, calcium channel blockers, angiotensin receptor blockers, and endothelin receptor antagonists were used at similar frequencies across both groups.

Additionally, modified Rodnan skin scores (median range: 16 in dippers vs. 15 in non-dippers; *p* = 0.775) did not differ significantly, indicating comparable severity of skin involvement.

## 4. Discussion

In this study, we demonstrated that the non-dipper blood pressure pattern was significantly more common in SSc patients compared to in the control group. The non-dipper blood pressure pattern, recognized as a significant indicator of cardiovascular risk, was identified in 83.9% of patients with SSc, whereas this pattern was present in only 25.8% of individuals in the control group. ABPM, conducted during two separate nighttime periods (24:00 to 04:00 and 04:00 to 08:00), revealed a consistent non-dipping pattern in SSc patients throughout the night. This observation indicates the widespread disruption of circadian autonomic regulation in SSc, affecting the entire nighttime duration instead of being limited to a specific phase. Moreover, the lack of a statistically significant variation in the occurrence of the non-dipper pattern between the two groups at different time intervals further supports the reliability of this finding.

As in the general population, fluctuations in BP according to the circadian rhythm in patients with HTN carry significant diagnostic and prognostic implications. The non-dipper pattern is observed in approximately 30–40% of hypertensive individuals, and this is an important risk indicator for increased cardiovascular morbidity and mortality.

In the pathophysiology of the non-dipper pattern, sympathetic nervous system activity throughout the night, circadian disruptions in cortisol secretion, the dysfunction of the renin–angiotensin–aldosterone system, and significant endothelial damage play a key role. In these individuals, mechanisms such as decreased nitric oxide (NO) production from endothelial cells and increased endothelin-1 (ET-1) levels cause the disruption of vascular tone regulation; as a result, arterial stiffness increases and the risk of target organ damage increases significantly. This process is associated with serious clinical outcomes such as left ventricular hypertrophy, microalbuminuria, ischemic stroke, and myocardial infarction. Sudden increases in BP, especially in the morning hours, may trigger ischemic events in non-dipper individuals. Hemodynamic stress occurring during the morning peak facilitates atherosclerotic plaque rupture and thrombosis formation, increasing the risk of acute cardiovascular events. Therefore, the non-dipper pattern is not only a circadian variation, but also a strategic parameter to be considered in clinical management [20,21].

Recent studies have revealed that the non-dipper pattern creates not only hemodynamic, but also immunological and metabolic effects. The levels of pro-inflammatory cytokines, such as TNF-α and IL-6, which tend to increase during the nighttime hours, have been found to correlate with circadian rhythm disturbances. This association suggests that, particularly in the context of autoimmune diseases such as SSc and rheumatoid arthritis, BP regulation is disrupted through multiple mechanisms [22,23].

In clinical practice, accurately identifying the non-dipper pattern is critical for individualizing treatment strategies. Within the framework of chronotherapy, the goal is to administer antihypertensive medications at times that align with the individual’s circadian blood pressure pattern. Antihypertensive treatments, especially given for nighttime regulation, may reduce the incidence of cardiovascular events by suppressing the risk of morning peak in non-dipper individuals [24]. In this context, the early detection of the non-dipper pattern contributes not only to the prediction of events, but also to the creation of individualized and timing-sensitive treatment plans.

Growing research indicates that individuals diagnosed with SSc tend to experience more pronounced endothelial impairment compared to healthy individuals [25]. Moreover, studies have consistently highlighted a heightened risk of atherosclerosis within this group, spanning from silent early signs to fully developed vascular pathologies, thereby exacerbating cardiovascular risks [26]. Significantly higher levels of vascular endothelial growth factor, an essential component in endothelial healing, have been reported more often in individuals with systemic sclerosis compared to healthy control subjects [27]. These pathological processes undermine vascular stability and are thought to significantly contribute to the development of hypertension by intensifying endothelial damage, promoting vascular structural changes, and driving fibrotic transformations [28]. In individuals with systemic sclerosis who also present with Raynaud’s phenomenon, plasma levels of thrombomodulin have been reported to be markedly higher. Additionally, the elevated concentrations of plasminogen activator inhibitor observed in early morning samples imply that disruptions in circadian rhythms might play a role in endothelial injury. Collectively, these findings emphasize the central importance of endothelial dysfunction and circadian rhythm disturbances in the pathophysiology of SSc [29].

Several recent studies have highlighted the link between circadian rhythms and immune system activity. These investigations have also shed light on the potential role of immune mechanisms in the development of HTN. Understanding the timing of immune activity could be pivotal for designing more precise and temporally optimized treatment approaches [30].

Research involving mouse models has shown that the infiltration of monocytes into muscle tissues, along with the mobilization of hematopoietic stem cells and neutrophils to the bone marrow, is most pronounced during the nighttime. This finding underscores the importance of circadian rhythms in regulating the movement and positioning of immune cells, with certain immune responses becoming more active during the night. The nocturnal surge in these immune processes suggests an intensified activation of immune-driven events like inflammation during this period [31]. Maeda et al.’s study revealed a significant increase in endothelin-1 (ET-1), a potent vasoconstrictor, in individuals diagnosed with SSc. Notably, ET-1 levels at midnight (24:00) were substantially higher in the SSc group compared to healthy controls. This finding may partially explain the high frequency of the non-dipper pattern observed in our study. These results indicate that vascular reactivity and endothelial dysfunction in SSc may exhibit circadian fluctuations, with a tendency to worsen during the night. The increased nighttime levels of ET-1 could contribute to intensified vasoconstrictive responses, potentially aggravating vascular complications in those affected by SSc [32]. This study did not demonstrate the typical nighttime reduction in blood pressure; instead, a higher occurrence of the non-dipper BP profile was detected. Compared to healthy individuals, patients with systemic sclerosis exhibited diminished left ventricular ejection fraction and elevated extracellular volume in cardiac assessments. These observations may indicate subclinical cardiac involvement and disrupted autonomic control, both of which are frequently linked to SSc [33]. The role of IL-17 in SSc has become a subject of investigation in further studies. Recent studies have shown that IL-17 not only influences immune cell function, but also plays a significant role in vascular dynamics and fibrotic mechanisms. Vascular thickening and endothelial dysfunction, characteristic features of SSc, have been shown to be exacerbated by IL-17. In addition to inducing the production of inflammatory mediators by endothelial cells, IL-17 is reported to play an important role in vascular pathology by regulating the proliferation and migration of vascular smooth muscle cells. The activation of these cells also contributes to fibrotic processes by promoting collagen production [34].

Individuals diagnosed with rheumatoid arthritis frequently report morning joint stiffness, a symptom that has been correlated with elevated circulating levels of pro-inflammatory cytokines such as tumor necrosis factor and interleukin-6 during the early morning hours [35]. This clinical manifestation is also believed to be influenced by increased sympathetic nervous system activity upon waking, which may contribute to morning elevations in blood pressure [36]. A study involving patients with rheumatoid arthritis demonstrated a diminished nocturnal decline in blood pressure relative to healthy controls, an effect attributed to underlying inflammatory processes [37]. Additionally, research involving individuals with SSc has shown that, compared to control subjects, they exhibit a higher augmentation index yet lower pulse wave velocity. These findings imply that distinct pathophysiological pathways may underline arterial stiffness and vascular function in the context of SSc [38].

Vascular dysfunction in SSc leads to a variety of clinical complications ranging from Reynaud’s phenomenon to pulmonary arterial hypertension. For this reason, agents that improve blood flow in the vascular area are frequently used in treatment strategies. Calcium channel blockers increase the digital blood flow and reduce ischemic attacks by providing peripheral vasodilation, especially in patients with Reynaud’s phenomenon [39]. Similarly, phosphodiesterase-5 inhibitors (PDE5is) both reduce Reynaud’s symptoms and relieve the load on the right heart by reducing pulmonary vascular resistance in patients with pulmonary arterial hypertension [40]. Endothelin receptor antagonists contribute to the improvement of vascular tone and the limitation of fibrosis by blocking the effect of endothelin-1 [41]. In our study, the higher frequency of CCB and PDE5is use in the non-dipper group indicates that vascular dysfunction may be more pronounced in this group. In addition, angiotensin receptor blockers (ARBs) are notable not only for their antihypertensive effects, but also for their endothelial-function-improving effects [42]. Targeted and timing-sensitive use of these agents in patients with a non-dipper pattern may be effective in preventing cardiovascular events by suppressing morning peaks.

In our study, a substantial proportion of patients with systemic sclerosis exhibited a non-dipper blood pressure pattern, indicating an absence of the expected nocturnal decline in blood pressure. This finding highlights the need for therapeutic strategies that extend beyond targeting fibrotic processes and also address vascular tone and autonomic regulation. Calcium channel blockers, commonly used in clinical practice, promote peripheral vasodilation, thereby reducing the frequency of Raynaud’s attacks. They also enhance digital blood flow during the night, which may help stabilize circadian blood pressure fluctuations. Similarly, phosphodiesterase type 5 inhibitors can reduce pulmonary vascular resistance and potentially limit nocturnal elevations in both pulmonary and systemic blood pressure. Endothelin receptor antagonists, used particularly in patients with elevated endothelin-1 levels, counteract vasoconstriction and contribute to a more physiologic blood pressure profile. Angiotensin receptor blockers offer multifaceted benefits by lowering blood pressure, improving endothelial function, and potentially limiting fibrosis progression. In our analysis, no significant differences were found in the use of these medications between patients with non-dipper and dipper blood pressure patterns. However, the more frequent use of angiotensin receptor blockers in patients with diffuse cutaneous systemic sclerosis may reflect a higher degree of vascular dysfunction in this subgroup. Taken together, these findings underscore the importance of further large-scale, prospective, and controlled studies to evaluate the impact of medication timing on blood pressure patterns in systemic sclerosis [41,42,43,44,45].

Our study has some limitations. First, the small sample size may limit the broader applicability of the findings. Studies involving a larger patient group are likely to enhance the reliability of the results. In addition, the fact that not all factors that may affect BP, such as the medical treatments of the patients included in the study, are the same, constitutes a limitation in terms of homogeneity. However, the fact that the participants had partially similar characteristics balanced this situation to some extent. Due to the small number of patients in the hypertensive group and the irregular timing of their medication intake, the type of antihypertensive drugs and the timing of their use were not analyzed. This may have influenced the 24 h blood pressure measurements in our study. Although 4 h periods at night offer an innovative method for evaluating circadian BP changes, more research is needed to determine whether these periods are ideal for each individual. The findings of this study reveal circadian BP changes and the presence of a non-dipper pattern in scleroderma patients. Future studies may provide important contributions to the treatment and follow-up strategies of these patients by examining the relationship between the non-dipper pattern and other variables in more detail in patients with scleroderma. In this context, determining blood pressure patterns in patients with SSc may lead to the development of an approach in which blood pressure treatment is synchronized with the patient’s circadian rhythm. This strategy could enhance treatment efficacy and be beneficial in follow-up, highlighting the potential for more personalized and effective disease management in this patient group.

## 5. Conclusions

In summary, the elevated occurrence of the non-dipper pattern among patients with systemic sclerosis underscores the importance of circadian rhythm disturbances within this group. Our study demonstrates that the distinct vascular and inflammatory abnormalities associated with SSc contribute not only to anatomical transformations, but also to notable changes in BP dynamics. Consequently, implementing 24 h ABPM as part of the routine clinical management of SSc patients could be instrumental in detecting cardiovascular risk factors early and preventing organ damage. Analyzing individual circadian BP trends can further refine treatment strategies and monitoring accuracy. Adopting this personalized approach has the potential to improve health outcomes and alleviate cardiovascular strain in this high-risk population.

## Figures and Tables

**Table 1:** Demographic characteristics and comorbidities of the systemic sclerosis (SSc) and non-SSc groups.

	Non-SSc Groups (*n* = 31)	SSc Groups (*n* = 31)	*p*
Age	52.2 ± 11.1	55.4 ± 10	0.234 *
Female Gender	27(87.1%)	29 (93.5%)	0.688
Hypertension	10 (32.3%)	11 (35.5%)	0.788
Body Mass Index (kg/m^2^)	25.8 ± 3.9	26.1 ± 4.2	0.762 *

* Student’s *t*-test, categorical variables were compared using Chi-squared test.

**Table 2:** Comparison of laboratory parameters of systemic sclerosis (SSc) and non-SSc groups.

	Non-SSc Groups (*n* = 31)	SSc Groups (*n* = 31)	*p*
Urea (mg/dL)	30 (26–34)	28 (24–37)	0.854 ^†^
Creatinine (mg/dL)	0.74 (0.69–0.84)	0.69 (0.58–0.77)	0.055 *
Sodium (mEq/L)	141 (138–141)	141 (139–142)	0.549 *
Potassium (mEq/L)	4.5 (4.3–4.8)	4.3 (4–4.5)	**0.013** ^†^
Aspartate aminotransferase (U/L)	15 (12–23)	18 (14–26)	0.113 ^†^
Alanine aminotransferase (U/L)	23 (17–30)	21 (15–28)	0.455 ^†^
Gamma-glutamyl transferase (U/L)	21 (17–30)	24 (16–38)	0.549 ^†^
Lactate dehydrogenase (U/L)	214 (183–234)	227 (193–252)	0.155 ^†^
Total bilirubin (mg/dL)	0.5 (0.4–0.7)	0.44 (0.32–0.74)	0.427 ^†^
Direct bilirubin (mg/dL)	0.1 (0.1–0.3)	0.1 (0.1–0.2)	0.872 ^†^
Hemoglobin (g/dL)	14 (13–14.9)	12 (11.6–12.9)	**<0.001** ^†^
Platelet (×10^9^/L)	234 (184–269)	246 (171–317)	0.730 ^†^
Leukocyte (×10^9^/L)	7.3 (5.9–9.4)	5.74 (4.76–6.87)	**0.002** ^†^
Low-density lipoprotein cholesterol (mg/dL)	119 (96–146)	113 (83–131)	0.260 *
Triglyceride (mg/dL)	119 (76–198)	102 (71–132)	0.240 ^†^
High-density lipoprotein cholesterol (mg/dL)	49 (43–57)	51 (39–62)	0.966 *
Total cholesterol (mg/dL)	186 (173–227)	176 (139–217)	0.248 *

^†^ Mann–Whitney U test, * Student’s *t*-test.

**Table 3:** Comparison of ambulatory blood pressure monitoring data of systemic sclerosis (SSc) and non-SSc groups.

	Non-SSc Groups (*n* = 31)	SSc Groups (*n* = 31)	*p*
Daytime Systolic blood pressure	124 (115–144)	121 (109–131)	0.102 ^†^
Daytime Diastolic blood pressure	76 (71–83)	68 (64–77)	**0.014** ^†^
Nighttime Systolic blood pressure	115 (103–125)	112 (104–126)	0.704 ^†^
Nighttime Diastolic blood pressure	64 (60–74)	61 (56–69)	0.086 ^†^
24 h Systolic blood pressure	123 (111–139)	119 (109–130)	0.310 ^†^
24 h Diastolic blood pressure	71 (68–79)	67 (62–75)	**0.031** ^†^
24:00–04:00 Systolic blood pressure	111 (100–126)	116 (103–126)	0.627 ^†^
24:00–04:00 Diastolic blood pressure	63 (60–74)	61 (52–68)	0.123 ^†^
04:00–08:00 Systolic blood pressure	114 (101–126)	116 (107–127)	0.455 *
04:00–08:00 Diastolic blood pressure	68 (61–74)	63 (57–72)	0.223 *

^†^ Mann–Whitney U test, * Student’s *t*-test.

**Table 4:** Comparison of dipping patterns of control and scleroderma groups according to time.

Time Period	Non-SSc Groups (*n* = 31)	SSc Patients (*n* = 31)	*p* (Between Groups) *
Day–Night Non-dipper blood pressure pattern	8 (25.8%)	26 (83.9%)	<0.001
24:00–04:00 Non-dipper blood pressure pattern	8 (25.8%)	22 (71.0%)	<0.001
04:00–08:00 Non-dipper blood pressure pattern	11 (35.5%)	25 (80.6%)	<0.001
Comparison between time intervals (within control group)	–	–	NS (*p* = 0.778) ’
Comparison between time intervals (within SSc group)	–	–	NS (*p* = 0.437) ’

* Chi-squared test was used to compare dipping frequencies between control and SSc groups for each time interval. Within-group comparisons (control and SSc, separately) were also analyzed using Chi-squared test. ’ NS: Not significant.

**Table 5:** Comparison of patients with diffuse and limited cutaneous systemic sclerosis scleroderma.

	Diffuse Systemic Sclerosis (*n* = 17)	Limited Cutaneous Systemic Sclerosis (*n* = 14)	*p* *
Day–Night Non-dipper blood pressure	15 (88.2%)	11 (78.6%)	0.467
24:00–04:00 Non-dipper blood pressure pattern	13 (76.5%)	9 (64.3%)	0.457
04:00–08:00 Non-dipper blood pressure pattern	15 (88.2%)	10 (71.4%)	0.239
Anti-SCL-70 (topoisomerase I)	13 (76.5%)	1 (7.1%)	**<0.001**
Anti-CENP-B (centromeric proteins)	0 (0%)	11 (78.6%)	**<0.001**
Anti-RNA-polymerase	4 (23.5%)	1 (7.1%)	0.217
Anti-RO-52	4 (23.5%)	1 (7.1%)	0.217
Anti-Rnp/Sm	4 (23.5%)	0 (0%)	0.052
Anti-JO-1	1 (5.9%)	0 (0%)	0.356
Anti-SS-A	3 (17.6%)	0 (0%)	0.098
Interstitial Lung Disease	12 (70.6%)	3 (21.4%)	**0.006**
Modified Rodnan Score	20 (5–24)	12 (2–20)	0.262
Pulmonary Hypertension	8 (47.1%)	9 (64.3%)	0.337
Reynaud’s Phenomenon	12 (70.6%)	12 (85.7%)	0.316
Calcium Channel Blocker	12 (70.6%)	13 (92.9%)	0.118
Phosphodiesterase-5 Inhibitor	6 (35.3%)	1 (7.1%)	0.062
Endothelin Receptor Antagonists	2 (11.8%)	1 (7.1%)	0.665
Angiotensin Receptor Blocker	8 (47.1%)	1 (7.1%)	**0.015**
Mycophenolate Mofetil	9 (52.9%)	2 (14.3%)	**0.025**
Azathioprine	6 (35.3%)	2 (14.3%)	0.183
Steroid	6 (35.3%)	2 (14.3%)	0.183
Hydroxychloroquine	9 (52.9%)	9 (64.3%)	0.524

* Chi-squared test was used for categorical variables. The numbers and percentages in the medication rows indicate the number and proportion of patients who were using the corresponding medication at the time of evaluation. Those written in bold are statistically significant.

**Table 6:** Comparison of autoantibody profiles, clinical features, and treatment regimens between Day–Night Dipper and Non-Dipper systemic sclerosis patients.

	Day–Night Dipper (*n* = 5)	Day–Night Non-Dipper (*n* = 26)	*p* *
Anti-SCL-70 (topoisomerase I)	2 (40%)	12 (46.2%)	0.800
Anti-CENP-B (centromeric proteins)	3 (60%)	8 (30.8%)	0.211
Anti-RNA-polymerase	1 (20%)	4 (15.4%)	0.797
Anti-RO-52	1 (20%)	4 (15.4%)	0.797
Anti-Rnp/Sm	0 (0%)	4 (15.4%)	0.347
Anti-JO-1	0 (0%)	1 (3.8%)	0.656
Anti-SS-A	0 (0%)	3 (11.5%)	0.424
Interstitial Lung Disease	2 (40%)	13 (50%)	0.682
Modified Rodnan Score	16 (2–22)	15 (4–22)	0.775
Pulmonary Hypertension	3 (60%)	14 (53.8%)	0.800
Reynaud’s Phenomenon	3 (60%)	21 (80.8%)	0.309
Calcium Channel Blocker	3 (60%)	22 (84.6%)	0.202
Phosphodiesterase-5 Inhibitor	0 (0%)	7 (26.9%)	0.187
Endothelin Receptor Antagonists	0 (0%)	3 (11.5%)	0.424
Angiotensin Receptor Blocker	2 (40%)	7 (26.9%)	0.555
Mycophenolate Mofetil	0 (0%)	11 (42.3%)	0.070
Azathioprine	3 (60%)	5 (19.2%)	0.056
Steroid	1 (20%)	7 (26.9%)	0.746
Hydroxychloroquine	2 (40%)	16 (61.5%)	0.371

* Chi-squared test was used for categorical variables. The numbers and percentages in the medication rows indicate the number and proportion of patients who were using the corresponding medication at the time of evaluation.

## Data Availability

The original contributions presented in this study are included in the article. Further inquiries can be directed to the corresponding author.

## Abbreviations

SSc—systemic sclerosis; BP—blood pressure; ABPM—ambulatory blood pressure monitoring; HTN—hypertension; ET-1—endothelin-1; SBP—systolic blood pressure; DBP—diastolic blood pressure; lcSSc—limited cutaneous systemic sclerosis; ACR—American College of Rheumatology; EULAR—European League Against Rheumatism; Anti-SCL-70—Anti-topoisomerase I antibody; Anti-CENP-B—Anti-centromere protein B antibody; Anti-RNA polymerase—Anti-RNA polymerase III antibody; Anti-RO-52—Anti-Ro52 autoantibody; Anti-Rnp/Sm—Anti-ribonucleoprotein/Smith antibody; Anti-JO-1—Anti-histidyl-tRNA synthetase antibody; Anti-SS-A—Anti-Sjögren’s syndrome-related antigen A antibody.
